# German translation, cross-cultural adaption and validation of the Venous Clinical Severity and Venous Disability Scores

**DOI:** 10.1186/s41687-023-00569-9

**Published:** 2023-03-15

**Authors:** Amun Hofmann, Julia Deinsberger, Philip Kienzl, Eva Rigler, Florian Anzengruber, Benedikt Weber

**Affiliations:** 1 Department of Dermatology, Medical University of Vienna, Vienna, Austria; 2 Department of Vascular and Endovascular Surgery, Klinik Ottakring, Montleartstraße 37, 1160 Vienna, Austria; 3 Department of Dermatology Zurich, Zurich, Switzerland

## Abstract

**Background:**

The Venous Clinical Severity Score (VCSS) and the Venous Disability Score (VDS) represent assessment tools for chronic venous disease (CVD) combining physician and patient reported outcomes. To date, German versions are not available. The present study aimed at translating the VCSS and VDS into German and validating the questionnaires.

**Methods:**

Translations of VCSS and VDS were compiled based on published guidelines considering potential differences in the use of German language in different countries. For validation, 33 patients with chronic venous disease and 5 healthy individuals were included in the pre-testing phase. Patients were examined twice by independent investigators to validate test–retest-validity culminating in 142 limb examinations. Internal consistency, inter-rater dependence and external reliability were subsequently evaluated.

**Results:**

All assessed metrics showed good internal consistency. Intra-class correlation coefficients were .75 for the VDS, .98 for the VCSS of the right leg and .90 for the VCSS of the left leg, indicating inter-rater independence. Furthermore, VCSS scores showed a modest positive correlation with CEAP C class and both VCSS and VDS showed a negative correlation with the physical component of the SF-12, indicating adequate external reliability.

**Conclusion:**

A pan-cultural German version of both the VCSS and VDS was established and validated as reliable tools to evaluate the severity of CVD in German speaking countries.

**Supplementary Information:**

The online version contains supplementary material available at 10.1186/s41687-023-00569-9.

## Introduction

Chronic venous insufficiency (CVI) represents one of the most common health care issues worldwide, significantly impairing the quality of life of affected patients [1]. Clinical symptoms include heaviness, pain, and pitting edema of the (lower) legs and, in advanced stages of disease, trophic changes of the skin, ranging from purpura jaune d'ocre to lipodermatosclerosis, white atrophy and, eventually, leg ulcers [2–5].

Diagnostic procedures primarily include physical examination and duplex sonography [6, 7]. Disease classification systems are frequently used to objectively evaluate the level of disease, for assessment of treatments, and for comparison and communication of scientific studies. The most commonly used classification scheme for CVI is the Clinical-Etiology-Anatomic-Pathophysiologic (CEAP) system [7], which is not only used in diagnosis and grading of CVI, but can also assist in establishing and assessing a treatment regimen [8, 9]. However, the lack of patient reported outcome measures (PROM) gave rise to the development of a disease score that unites physician and patient reported outcomes, the disease severity scores Venous Clinical Severity Score (VCSS) and the Venous Disability Score (VDS), which were first published in 2000 along with the Venous Segmental Disease Score as part of the Venous Severity Scoring System [10]. Contrary to the CEAP score, VCSS and VDS also include information on the subjective disease burden on the patient.

The VCSS consists of patient reported elements assessed during the structured interview section, including pain and edema, as well as clinician reported elements evaluated during the clinical examination, including the extent of varicose veins, hyperpigmentation, inflammation, and the presence of ulcerations [10]. The VDS quantifies the patient reported degree of disability caused by CVI in daily routine. Both tools are widely accepted and validated tools to assess health-related quality of life and disease burden in CVI patients. However, the absence of a validated German version limits their use in German-speaking populations. Since the application depends on the English language skills and individual translation of the attending physician, objectivity and comparability is limited. German is the (co-)official language of five European countries and several regions and provinces such as South Tyrol, Italy, with roughly 100 million native speakers globally [11]. Considering this high number of German native speakers and the high prevalence of CVI the need for a validated tool to quantify disease severity in German-speaking CVI patients is evident [1].

A standardized approach to cross-cultural adaption and translation of tools to assess disease severity has previously been discussed and guidelines were developed [12–14]. In addition to the proposed guidelines, the cultural and geographical heterogeneity of German speakers must not be neglected during the process of translation. Therefore, Austrian, German, and Swiss natives were involved in the development of a pan-cultural German translation and the validation of the derived German VCSS and VDS.

## Methods

The study was designed and conducted in line with the Declaration of Helsinki [15]. Approval was issued by the Ethics Committee of the Medical University of Vienna (Identifier: 2158/2019). All participants gave their written informed consent prior to inclusion in the study.

### Development of a German translation

**Fig. 1:**
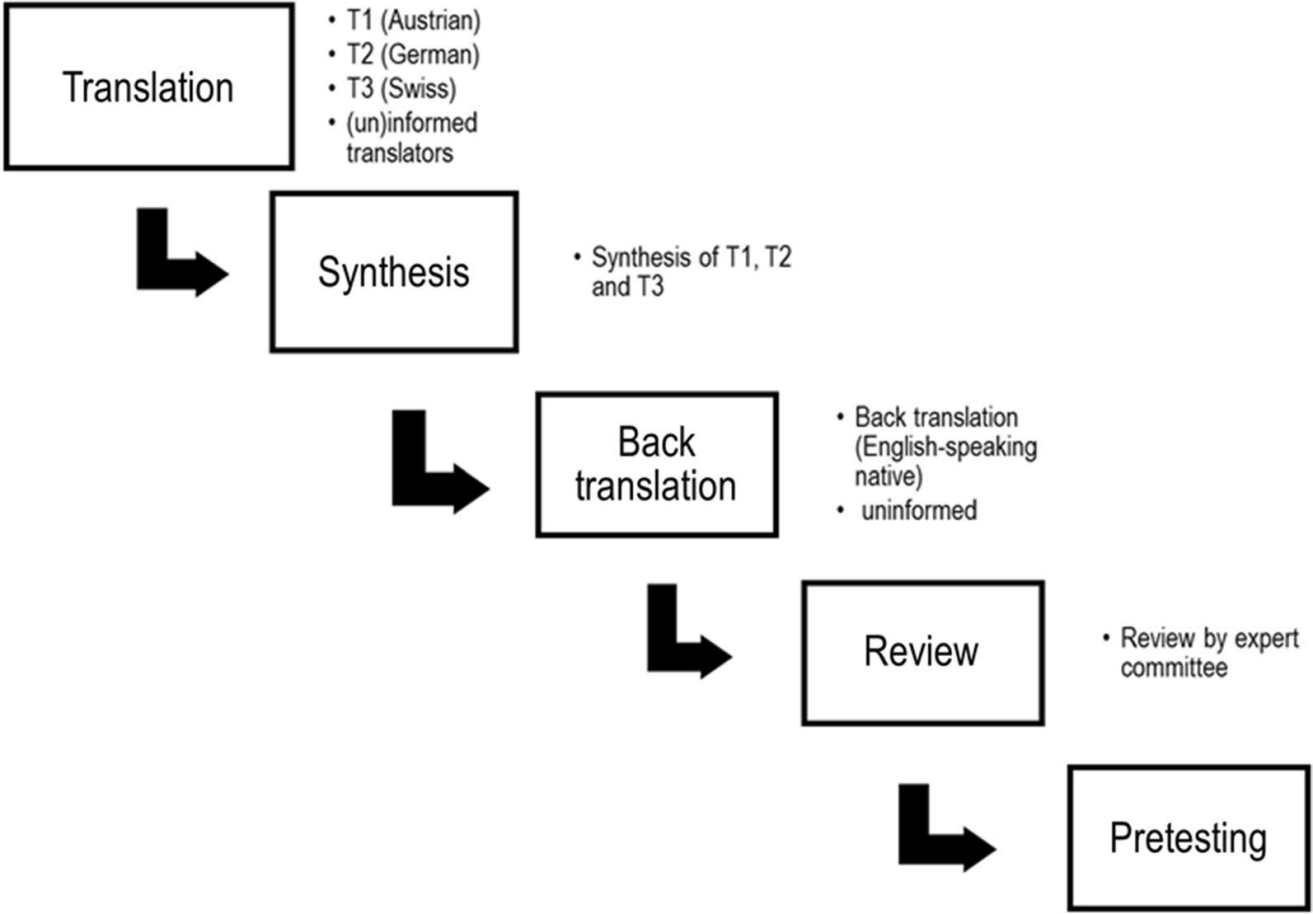
Flowchart illustrating the translation and validation process

The present study followed an adapted version of the Guidelines for the Process of Cross-Cultural Adaptation of Self-Report Measures by Beaton et al. [14] to establish a German translation of the VCSS and VDS. The original venous clinical severity Score from 2000 [10] was used instead of its revised version from 2010 [16], based on the clinical experience of involved experts and due to the higher number of citations of the original version (64 vs. 39 citations since 2010, PubMed search conducted on 09.07.2022). Comparability in-between clinical studies is therefore better using the original version. Three translations were carried out, each by German native-speakers (first language) from one of the three culturally distinguishable German-speaking countries (Austria, Germany, and Switzerland). All three collaborators are additionally fluent in English. Two out of three translators (Austrian and German) had no medical background. Translations were merged by the developer to result in a pre-final translation. The developer was a native Austrian medical doctor. Subsequently, a back translation was performed by an English native speaker (non-medical and not familiar with the original scores) that was compared to the original version and further reviewed by an expert committee consisting of German native health professionals (2 Austrian and 1 Swiss). The reviewed version was forwarded to the developer, who produced a final translation, taking suggestions of the committee into account (Fig. 1).

### Validation of the German VCSS and VDS

Patients were recruited at the phlebology outpatient clinic and the inpatient ward of the Department of Dermatology at the Medical University of Vienna. Study participation consisted of two study visits. The first study visit consisted of the completion of the German VCSS and VDS and a German Short Form 12 Health Survey (SF-12). CEAP classification was evaluated by the investigator. The second study visit consisted of another completion of the German VCSS and VDS. The second visit took place within 1–30 days of the first study visit and was carried out by another independent blinded clinician. Assessments were conducted by residents from the dermatological department. Participation in the study did not affect further diagnostic procedures and treatment. Based on Beaton et al., we aimed at evaluating the translations in 30 patients [14]. To evaluate specificity, the established tools were additionally assessed in 5 healthy volunteers. The validation did not include any qualitative measures due to the clinician assessed nature of the tools.

### Statistical analysis

Descriptive statistics were calculated for general demographic information (participant age and sex). For VCSS and VDS results, means, standard deviations and quantiles were calculated. Internal consistency/reliability was assessed through coefficient alpha (also known as Cronbach’s alpha), (Revelle’s) omega total and greatest lower bound. The intra-class correlation coefficient was used to measure inter-rater reliability in a test–retest setting. Pearson correlation coefficient was calculated to analyze external reliability/validity, correlating VCSS and VDS results to CEAP C-class and SF-12 results. In order to calculate correlation metrics for categorical CEAP C, classes were recoded based on their integer value plus .0 for “a”, .3 for “b”, .5 for “s” and .6 for “c” suffixes and subsequently treated as continuous variables. In the case of the SF-12 correlation analysis, VCSS and VDS mean refer to the arithmetic mean of the two examinations conducted by independent raters. Statistics were performed using R version 4.1.2 (R Foundation for Statistical Computing, Vienna, Austria).

## Results

### Translation

In general, the primary translations were quite homogenous in content. Differences in phrasing were found to be more stylistic than due to linguistic background. The VDS translation process did not feature particular difficulties. However, regarding the VCSS translation the uninformed translators had to consult medical dictionaries regarding specific anatomic and medical vocabulary such as *greater saphenous* or *induration*. This was not deemed to be of further concern since the questionnaire should be conducted by a health professional capable of explaining such terms to patients. Difficulties arose from the term *sliding scale* in the instructions of the original instrument. Eventually, the term was dismissed taking into account the informed translation conducted by a Dermatologist and the review of the expert committee, since it was not believed to increase comprehensibility of the translation while posing a risk for confusion. The translated scores are shown in Tables 1 and 2, printable versions including translated instructions are available in the Additional file 1: Table 1.

**Table 1:** German version of the venous clinical severity Score used during pre-testing

Merkmal	Nicht vorhanden = 0	Leicht = 1	Mittel = 2	Schwer = 3
Schmerz	Keiner	Gelegentlich, keine Einschränkung der Aktivitäten oder Bedarf an Schmerzmitteln	Täglich, leichte Einschränkung der Aktivitäten, gelegentlicher Bedarf an Schmerzmitteln	Täglich, starke Einschränkung der Aktivitäten oder regelmäßiger Bedarf an Schmerzmitteln
Krampfadern	Keine	Wenige, vereinzelt: retikuläre Venen	Mehrere: VSM Krampfadern Auf die Wade oder den Oberschenkel beschränkt	Ausgedehnt: Ausbreitung auf den Oberschenkel *und* die Wade oder VSM *und* VSP Beteiligung
Stauungsödem	Keines	Nur abendliches Knöchelödem	Ödem am Nachmittag, oberhalb der Knöchel	Morgendliches Ödem oberhalb der Knöchel, Änderung der Aktivitäten und Hochlagerung erforderlich
Hautpigmentierung	Keine oder herdförmig, leichte Intensität (Bräunung)	Diffus, aber regional limitiert und alt (braun)	Diffus über Großteil des Unterschenkels verteilt (unteres Drittel) *oder* frische Pigmentierung (rötlich-livide)	Größere Ausbreitung (oberhalb des unteren Drittels) *und* frische Pigmentierung
Entzündung	Keine	Leichte Weichteilentzündung, Randbereich des Ulkus begrenzt	Moderate Weichteilentzündung, Großteil des Unterschenkels betreffend (unteres Drittel)	Starke Weichteilentzündung (unteres Drittel und darüber) oder signifikantes venöses Ekzem
Verhärtung	Keine	Herdförmig, rund um den Knöchel (< 5 cm)	Medial oder lateral, weniger als das untere Drittel des Beines	Gesamtes unteres Drittel des Beines oder mehr
Anzahl aktiver Ulzerationen	0	1	2	> 2
Dauer aktiver Ulzerationen	Keine	< 3 Monate	> 3 Monate, < 1 Jahr	> 1 Jahr nicht verheilt
Größe aktiver Ulzerationen	Keine	< 2 cm Durchmesser	2 bis 6 cm Durchmesser	> 6 cm Durchmesser
Kompressionstherapie	Keine in Verwendung oder incompliant	Unregelmäßiger Gebrauch von Strümpfen	Tragen elastischer Strümpfe an den meisten Tagen	Volle Compliance: Strümpfe und Hochlagerung

**Table 2:** German version of the venous disability score used in pre-testing

0 = asymptomatisch
1 = symptomatisch aber in der Lage übliche Aktivitäten* ohne Kompressionstherapie auszuführen
2 = kann übliche Aktivitäten nur mit Kompressionstherapie und/oder Hochlagerung der Gliedmaßen ausführen
3 = nicht in der Lage übliche Aktivitäten* auszuführen, selbst unter Kompressionstherapie und/oder Hochlagerung der Beine

### Patient characteristics

The derived translated VCSS and VDS were evaluated in 33 and 31 patients with confirmed chronic venous insufficiency respectively, and in 5 healthy individuals. Demographic information of the patients is depicted in Table 3.

**Table 3:** Demographics of the study population

Characteristic	Study population (n = 33)
*Age group*	
20–39 years	6 (18.2%)
40–49 years	9 (27.3%)
50–59 years	5 (15.2%)
60–69 years	4 (12.1%)
70–79 years	5 (15.2%)
> 80 years	4 (12.1%)
*Sex*	
Female	16 (48.5%)
Male	17 (51.5%)

**Fig. 2:**
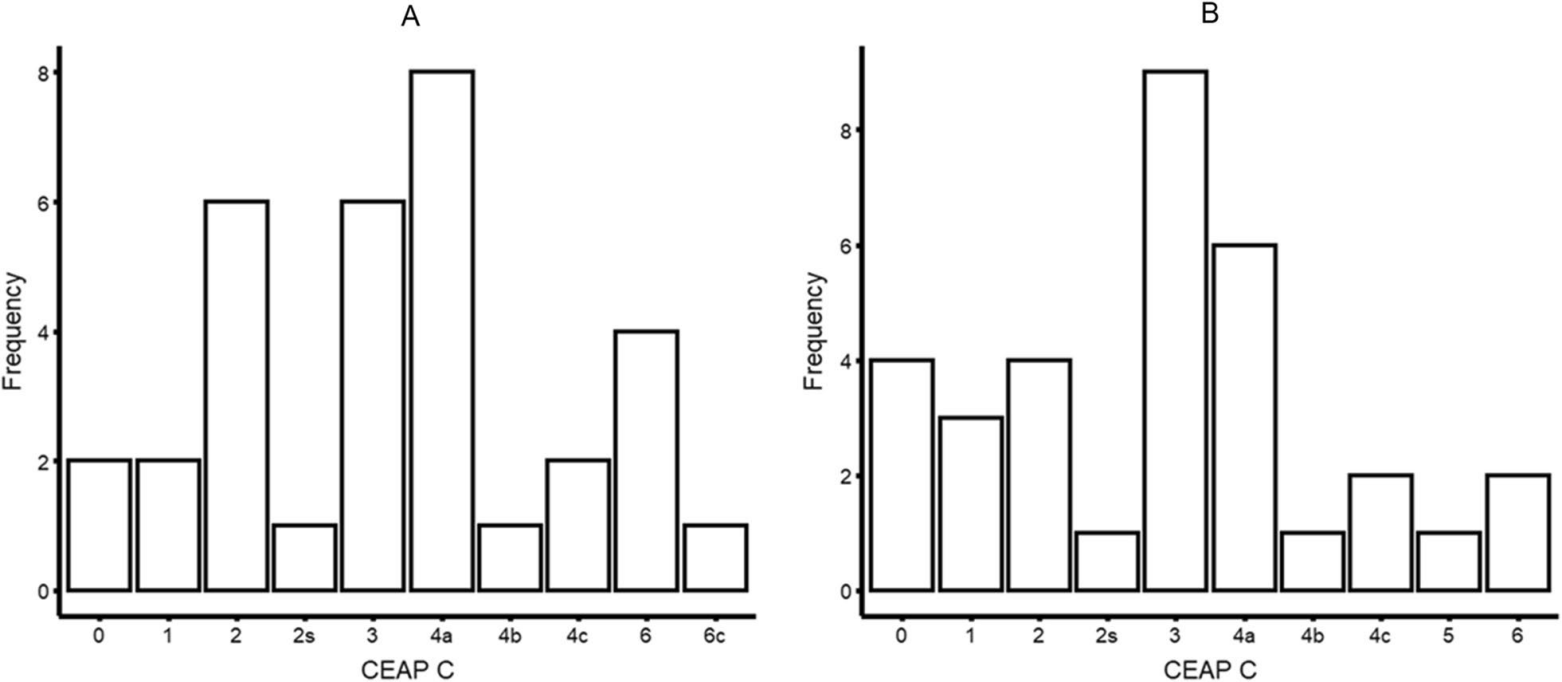
CEAP C class in the study population. **A** right leg, **B** left leg. C0 = no evidence of venous disease, C1 = Teleangectasia, C2 = Varicose veins, C3 = Edema, C4 = Skin changes, C5 = Healed venous ulcer, C6 = Active venous ulcer

The clinical score (C class) of the CEAP classification grades chronic venous insufficiency from C0, which means no venous disease to C6, describing the presence of an active venous leg ulcer. Distribution of CEAP C class in the study population is shown in Fig. 2. CEAP C class was graded C0 in 6 (9.1%), C1 in 5 (7.6%), C2 in 10 (15.2%), C2s in 2 (3.0%), C3 in 15 (22.7%), C4a in 14 (21.2%), C4b in 2 (3.0%), C4c in 4 (6.1%), C5 in 1 (1.5%), C6 in 6 (9.1%) and C6c in 1 (1.5%) limb.

### Validation of the translated VCSS and VDS

Measures of central tendency and dispersion scored during validation stratified by limb and timepoint are exhibited in Table 4. Interpretation of Cronbach’s alpha based on George and Mallery [17] is *Acceptable* in case of the first examination of the left leg and *Good* in the remaining three instances. All metrics indicate high reliability (Table 5).

**Table 4:** Measures of central tendency and dispersion scored during validation of the translated VCSS

Test	Mean	Standard deviation	Median	Q1–Q3
VCSS right leg 1	7.8	5.5	6	5–10
VCSS right leg 2	8.2	5.8	7	5–10
VCSS left leg 1	6.6	4.5	5	4–8
VCSS left leg 2	7.1	5.4	5	4–9

1 = first examination, 2 = second examination

**Table 5:** Assessment of internal consistency of the translated VCSS. 1 = first examination, 2 = second examination

Test	Cronbach’s alpha	Revelle’s omega total	Greatest lower bound	Alpha interpretation
VCSS right leg 1	.83	.91	.94	Good
VCSS right leg 2	.87	.94	.95	Good
VCSS left leg 1	.77	.90	.91	Acceptable
VCSS left leg 2	.81	.92	.93	Good

The intra-class correlation coefficients (ICC) for the German VCSS and VDS showed overall satisfactory inter-rater agreement, which indicates that the results are independent of the investigator (Table 6, Additional file 2: Figures S1 and S2).

**Table 6:** Inter-rater agreement measures

Test	ICC (95% CI)	Interpretation based on Cicchetti [31]	Interpretation based on Koo and Li [32]
VDS	.75 (.54–.87)	Excellent	Good
VCSS right leg	.98 (.96–.99)	Excellent	Excellent
VCSS left leg	.90 (.81–.95)	Excellent	Good

*ICC* intra-class correlation coefficient

**Fig. 3:**
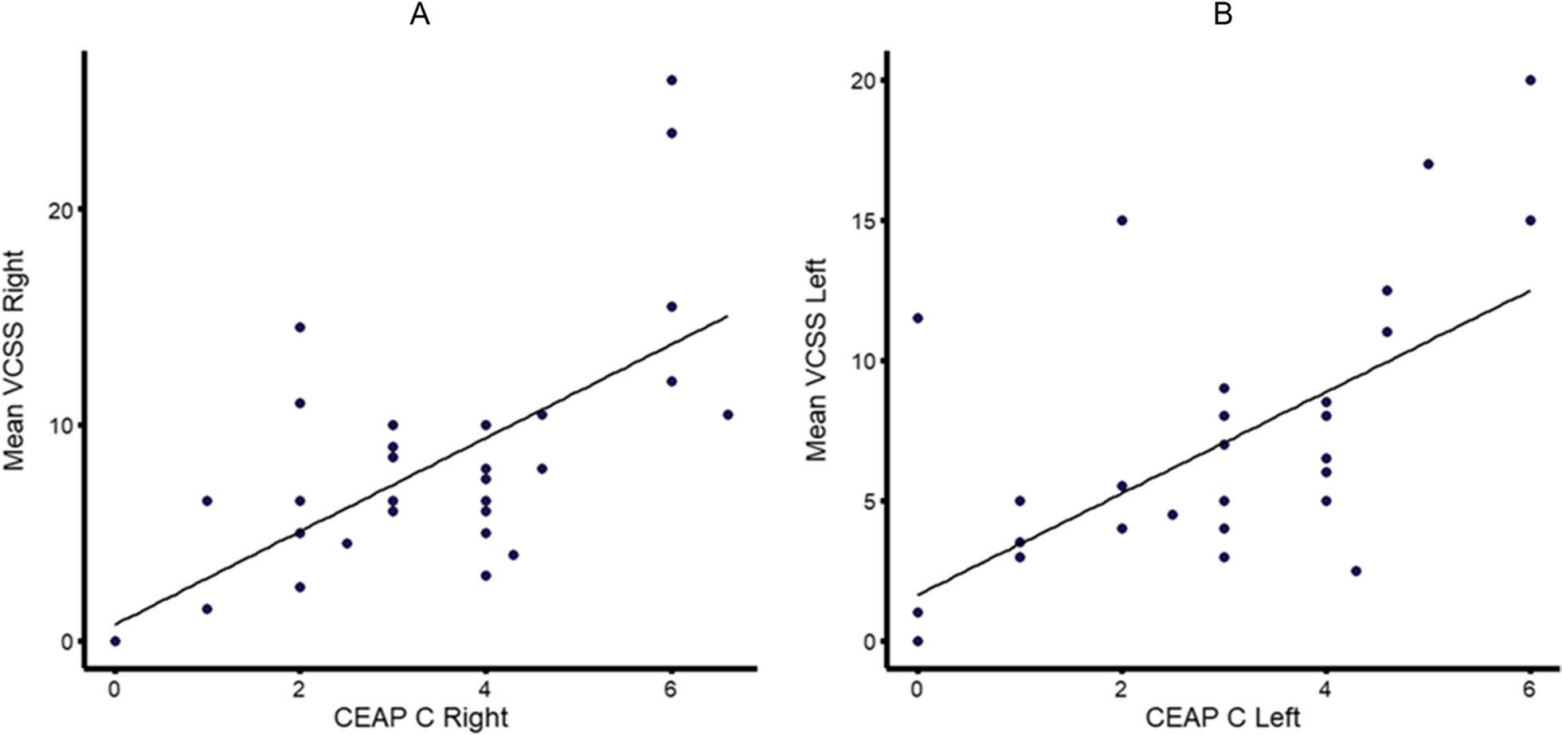
Correlation of VCSS score and CEAP C class **A** right leg, **B** left leg

To evaluate external reliability, the correlation of VCSS results and CEAP C class was assessed. Pearson correlation coefficient amounted to 0.65 (95% CI .39–.81) and .61 (95% CI .34–.79) for the right and left leg respectively (Fig. 3). Correlation of the physical component of the SF-12 and the mean VDS and mean VCSS (both legs combined) resulted in a negative correlation (Pearson’s r; − .37 (95% CI − .65 to− .02) and − .49 (95% CI − .72 to − .17)), i.e., higher VCSS and VDS scores were associated with reduced physical well-being.

In order to examine the specificity of the translated VCSS and VDS, the instruments were applied to 5 healthy subjects resulting in a score of 0 in all instances.

## Discussion

Chronic venous disease ranks among the most frequent health care issues worldwide affecting approximately 90% of the population in developed countries [18]. It encompasses a wide range of morphologic changes and functional abnormalities ranging from telangiectasias to the development of venous leg ulcers [8]. Risk factors for progression to chronic venous insufficiency include age and obesity. Thus, which an aging population and a growing prevalence of obesity, the burden of CVD is expected to increase [19].

Assessment tools are used to evaluate the severity of disease, to provide for standardized evaluation, and to facilitate comparability of treatment outcome. The VCSS and VDS are widely distributed and thoroughly validated tools for CVD that combine patient reported items and clinical assessment [20]. The VDS reflects CVD-associated limitations in everyday life while the VCSS is a 10-item questionnaire scoring clinical signs and symptoms of the disease. However, to date no German version has been published or validated for clinical use. Hence, the application of the English tools depends on individual translation of the attending physician and comparability of different studies in German-speaking countries is therefore limited due to inherent bias. The distorting effects of non-standardized translations on outcomes in clinical practice and adjacent studies have been discussed previously [21–23].

Therefore, pan-cultural German translations of the VCSS and VDS were established based on international guidelines for cross-cultural translations [14] and subsequently validated in a sample of CVD patients. The primary translations included informed and uninformed translators from Austria, Germany, and Switzerland, which were merged, and back-translated while being assessed by a developer and an expert committee at every stage of production. For validation, VCSS was performed separately for each leg in 33 patients with chronic venous disease out of which 31 also completed the VDS. Investigations were performed twice by two independent investigators. The German instruments proved to be reliable measures of disease severity showing an adequate internal consistency and excellent inter-rater reliability. VCSS scores showed a strong positive correlation to CEAP C class and both showed a negative correlation to SF-12 scores confirming external validity of the translated versions. Furthermore, to investigate the specificity VCSS and VDS were evaluated in 5 healthy individuals, whereof all showed scores of 0.

However, the proposed and investigated translation was only validated in Austrian patients so far. While the translation process was pan-cultural, the preliminary uni-cultural application needs further validation in Swiss and German patients before it should be considered finalized. Printable versions as used during pre-testing and for future clinical application of the translated VCSS and VDS are included in the online supplement. (Additional file 1).

Recent translations of the VCSS included Mandarin [24], Hindi, and Marathi [25] indicating that the use of translated versions is of growing global interest. While other CVD scores such as the Aberdeen Varicose Vein Questionnaire are available in languages such as Dutch [26], Hungarian [27] or Portuguese [28], the application of the VCSS and VDS in European cohorts is still limited to the original version. The present study might induce the re-evaluation of establishing and validating translated versions of VCSS and VDS considering the globally high prevalence of chronic venous disease [29, 30].

## Conclusion

In summary, we hereby present pan-cultural German versions of both the VCSS and VDS that were validated as reliable tools to evaluate the severity of chronic venous disease.

## Supplementary Information

**Additional file 1.** Venous Clinical Severity Score and Venous Disability Score.

**Additional file 2**.** Figure S1**. Bland-Altman plots of the investigated scores. A) VCSS right leg, B) VCSS left leg, C) VDS.** Figure S2**: VCSS scores of the first and second examination including linear regression trendlines. A) VCSS right leg, B) VCSS left leg.

## Data Availability

Data will be made available upon reasonable request to the corresponding author.
