# Supplementary material for: German translation, cross-cultural adaption and validation of the Venous Clinical Severity and Venous Disability Scores
Source: J Patient Rep Outcomes. 2023 Mar 15;7:28. doi: 10.1186/s41687-023-00569-9 (PMC10017906; doi:10.1186/s41687-023-00569-9)
Supplement: Supplementary file 1 — Additional file 1. Venous Clinical Severity Score and Venous Disability Score. [file 41687_2023_569_MOESM1_ESM.pdf]

Venous Clinical Severity Score

Merkmal	Nicht vorhanden = 0	Leicht = 1	Mittel = 2	Schwer = 3
Schmerz	Keiner	Gelegentlich, keine Einschränkung der Aktivitäten oder Bedarf an Schmerzmitteln	Täglich, leichte Einschränkung der Aktivitäten, gelegentlicher Bedarf an Schmerzmitteln	Täglich, starke Einschränkung der Aktivitäten oder regelmäßiger Bedarf an Schmerzmitteln
Krampfadern*	Keine	Wenige, vereinzelt: retikuläre Venen	Mehrere: VSM Krampfadern Auf die Wade oder den Oberschenkel beschränkt	Ausgedehnt: Ausbreitung auf den Oberschenkel *und* die Wade oder VSM *und* VSP Beteiligung
Stauungsödem†	Keines	Nur abendliches Knöchelödem	Ödem am Nachmittag, oberhalb der Knöchel	Morgendliches Ödem oberhalb der Knöchel, Änderung der Aktivitäten und Hochlagerung erforderlich
Hautpigmentierung‡	Keine oder herdförmig, leichte Intensität (Bräunung)	Diffus, aber regional limitiert und alt (braun)	Diffus über Großteil des Unterschenkels verteilt (unteres Drittel) *oder* frische Pigmentierung (rötlich-livide)	Größere Ausbreitung (oberhalb des unteren Drittels) *und* frische Pigmentierung
Entzündung	Keine	Leichte Weichteilentzündung, Randbereich des Ulkus begrenzt	Moderate Weichteilentzündung, Großteil des Unterschenkels betreffend (unteres Drittel)	Starke Weichteilentzündung (unteres Drittel und darüber) oder signifikantes venöses Ekzem
Verhärtung	Keine	Herdförmig, rund um den Knöchel (< 5 cm)	Medial oder lateral, weniger als das untere Drittel des Beines	Gesamtes unteres Drittel des Beines oder mehr
Anzahl aktiver Ulzerationen	0	1	2	> 2
Dauer aktiver Ulzerationen	Keine	< 3 Monate	> 3 Monate, < 1 Jahr	> 1 Jahr nicht verheilt
Größe aktiver Ulzerationen§	Keine	< 2 cm Durchmesser	2 bis 6 cm Durchmesser	> 6 cm Durchmesser
Kompressionstherapie‖	Keine in Verwendung oder incompliant	Unregelmäßiger Gebrauch von Strümpfen	Tragen elastischer Strümpfe an den meisten Tagen	Volle Compliance: Strümpfe und Hochlagerung

*: Der Durchmesser der Krampfadern muss >4mm sein um eine Abgrenzung von C1 zu C2 zu gewährleisten.

†: Annahme eines venösen Ödems basierend auf den Eigenschaften (z.B. derbes [statt teigig oder schwammig] Ödem), mit signifikantem Einfluss von Stehen/ Hochlagern der Extremität und/ oder anderen klinischen Zeichen venöser Ätiologie (z.B. Krampfadern, tiefe Venenthrombose in der Anamnese). Das Ödem muss regelmäßig auftreten (z.B. täglich). Gelegentliche oder leichte Ödeme sind hier zu vernachlässigen.

‡: Herdförmiger Pigmentierung über einer Krampfader qualifiziert hierfür nicht

§: Größte Dimension/ Ausdehnung des größten Ulkus

‖: Die Patientengeschichte bezüglich einer Kompressionstherapie ist hier miteinzubeziehen.

VSM = Vena saphena magna, VSP = Vena saphena parva

Venous Disability Score

0 = asymptomatisch

1 = symptomatisch aber in der Lage übliche Aktivitäten* ohne Kompressionstherapie auszuführen

2 = kann übliche Aktivitäten nur mit Kompressionstherapie und/oder Hochlagerung der Gliedmaßen ausführen

3 = nicht in der Lage übliche Aktivitäten* auszuführen, selbst unter Kompressionstherapie und/oder Hochlagerung der Beine

*: Übliche Aktivitäten: Aktivitäten des Patienten vor Beginn der Einschänkung durch die venöse Erkrankung
