# Supplementary material for: German translation, cross-cultural adaption and validation of the Venous Clinical Severity and Venous Disability Scores
Source: J Patient Rep Outcomes. 2023 Mar 15;7:28. doi: 10.1186/s41687-023-00569-9 (PMC10017906; doi:10.1186/s41687-023-00569-9)
Supplement: Supplementary file 2 — Additional file 2. Figure S1. Bland-Altman plots of the investigated scores. A) VCSS right leg, B) VCSS left leg, C) VDS. Figure S2: VCSS scores of the first and second examination including linear regression trendlines. A) VCSS right leg, B) VCSS left leg. [file 41687_2023_569_MOESM2_ESM.docx]

## Supplement

**
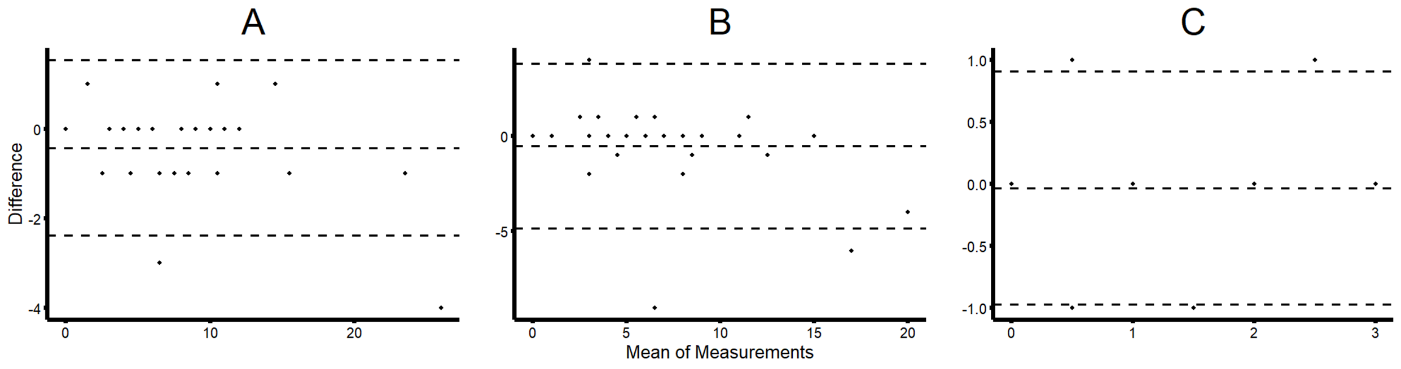
Figure S1.** Bland-Altman plots of the investigated scores. A) VCSS right leg, B) VCSS left leg, C) VDS


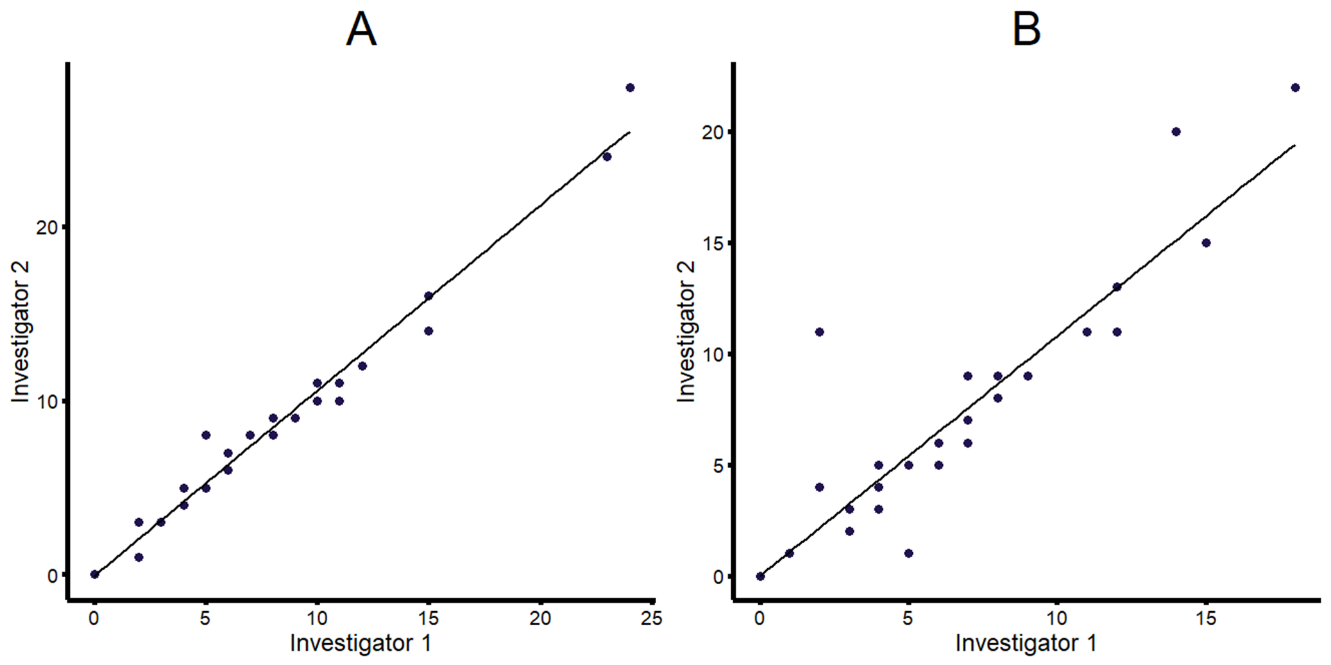
**Figure S2.** VCSS scores of the first and second examination including linear regression trendlines. A) VCSS right leg, B) VCSS left leg.
